# Bee venom attenuates neurodegeneration and motor impairment and modulates the response to L-dopa or rasagiline in a mice model of Parkinson’s disease

**DOI:** 10.22038/ijbms.2020.46469.10731

**Published:** 2020-12

**Authors:** Hanaa MM Badawi, Rania M Abdelsalam, Omar ME Abdel-Salam, Eman R Youness, Nermeen M Shaffie, Ezz-El Din S Eldenshary

**Affiliations:** 1 Holding Company for Biological Products, Vaccines and Drugs (VACSERA), Cairo, Egypt; 2 Department of Pharmacology and Toxicology, Faculty of Pharmacy, Cairo University, Cairo, Egypt; 3 Department of Toxicology and Narcotics, National Research Centre, Cairo, Egypt; 4 Department of Medical Biochemistry, National Research Centre, Cairo, Egypt; 5 Department of Pathology, National Research Centre, Cairo, Egypt

**Keywords:** Bee venom, Dopamine, Oxidative stress, Parkinson’s disease, Rotenone

## Abstract

**Objective(s):**

This study aimed to investigate the effect of bee venom, a form of alternative therapy, on rotenone-induced Parkinson’s disease (PD) in mice. Moreover, the possible modulation by bee venom of the effect of L-dopa/carbidopa or rasagiline was examined.

**Materials and Methods:**

Rotenone (1.5 mg/kg, subcutaneously; SC) was administered every other day for two weeks and at the same time mice received the vehicle (DMSO, SC), bee venom (0.065, 0.13, and 0.26 mg/kg; intradermal; ID), L-dopa/carbidopa (25 mg/kg, intraperitoneal; IP), L-dopa/carbidopa+bee venom (0.13 mg/kg, ID), rasagiline (1 mg/kg, IP) or rasagiline+bee venom (0.13 mg/kg, ID). Then, wire hanging and staircase tests were performed and mice were euthanized and brains’ striata separated. Oxidative stress biomarkers namely, malondialdehyde (MDA), nitric oxide (NO), reduced glutathione (GSH), paraoxonase-1 (PON-1), and total antioxidant capacity (TAC) were measured. Additionally, butyrylcholinesterase (BuChE), monocyte chemoattractant protein-1 (MCP-1), tumor necrosis factor-alpha (TNF-α), and dopamine (DA) were evaluated. Brain histopathological changes and caspase-3- expression were done.

**Results:**

Bee venom significantly enhanced motor performance and inhibited rotenone-induced oxidative/nitrosative stress, observed as a reduction in both MDA and NO along with increasing GSH, PON-1, and TAC. Besides, bee venom decreased MCP-1, TNF-α, and caspase-3 expression together with an increase in BuChE activity and DA content.

**Conclusion:**

Bee venom alone or in combination with L-dopa/carbidopa or rasagiline alleviated neuronal degeneration compared with L-dopa/carbidopa or rasagiline treatment only. Bee venom via its antioxidant and cytokine reducing potentials might be of value either alone or as adjunctive therapy in the management of PD.

## Introduction

Parkinson’s disease (PD) is a chronic neurodegenerative disease occurring in the elderly. PD is due to a preferential and progressive loss of the pigmented dopaminergic neurons in the substantia nigra pars compactica (SNc) that project into the striatum. The substantial dopaminergic loss that occurs in PD results in disruption of this basal ganglia circuitry and consequent corticostriatal imbalance together with the development of the cardinal features of the disease, i.e., the slowing of voluntary movements or bradykinesia, muscular rigidity, postural instability, gait disturbances, and hand tremor (1). There are also non-motor manifestations that include depression, cognitive changes, psychiatric symptoms as well as autonomic disturbances. The slow nigral cell death that occurs in PD is largely thought to be the result of oxidative damage and neuroinflammatory events that are triggered by environmental toxins and is likely to require genetic susceptibility as well (2). Several rodent studies indicated that the pesticide rotenone is capable of evoking oxidative nigrostriatal cell death and pathological (alpha-synuclein-like deposits) and motor changes similar to those occurring in the idiopathic form of PD, thereby strengthening a role for pesticide in its causation (3).

Bee venom is extracted from honey bees and is usually used in oriental medicine. It has been used to treat many diseases as chronic rheumatoid arthritis or osteoarthritis, skin diseases, and cancer. Bee venom possesses anti-inflammatory, anti-bacterial, antimutagenic, radioprotective, anti-nociceptive immunity promoting, hepatocyte protective, and anti-cancer activity. Also, it improves motor function and reduces motor neuron death in the spinal cord and restores normal neurotransmitter signaling (4-6). 

Apis mellifera venom has been identified as a complex combination of active peptides composed of melittin, phospholipase A_2_, apamin, adolapin, cardiopep, peptide 401, mast cell-degranulating peptide, and several enzymes, as phospholipase A_2_ (PLA_2_), phospholipase B, hyaluronidase, acid phosphatase, and alpha-glucosidase, in addition to different classes of enzymes in BV, as esterases, proteases and peptidases, protease inhibitors, and others (6, 7). Apamin, melittin, and phospholipase A_2 _have neuroprotective effects (8, 9), and adolapin has an anti-inflammatory potential (10). So BV and its components have immuno-modulatory and neuro-protective potentials and thus can be used for the treatment of neurodegenerative diseases (11).

The mainstay of the pharmacological treatment of PD is the dopamine precursor levodopa (L-3, 4-dihydroxy-phenylalanine), administered in combination with the peripheral decarboxylase inhibitor carbidopa which inhibits the peripheral metabolism of levodopa, thereby allowing its therapeutic concentrations to be achieved in the brain without disabling peripheral adverse effects (12). Rasagiline is a selective MAO-B inhibitor that is used in the treatment of PD with the aim of decreasing dopamine catabolism, thereby, increasing its brain levels. The drug is shown to improve motor performance when used as monotherapy in mildly symptomatic PD patients or as adjunctive therapy (13). These agents and other drugs available provide only symptomatic relief but their efficacy declines over time owing to the progressive nature of the disease with few dopaminergic cells remaining for dopaminergic stimulation (14, 15). Hence, the need for other drugs or therapeutic interventions to halt disease progression.

Recently, a number of experimental and clinical studies pointed out to a possible therapeutic value of bee venom in PD (16-20). In the 1-methyl-1,2,3,6-tetrahydropyridine (MPTP)-induced PD in mice, bee venom acupuncture protected against the loss of tyrosine hydroxylase in the striatum and substantia nigra (16, 17). Improvements in the hypokinetic motor behavior were also shown following bee venom injection in the 6-hydroxydopamine-induced PD (18). *In vitro*, bee venom application protected dopaminergic neurons from death due to rotenone (19). In patients with idiopathic PD, greater improvement in motor scale in the bee venom group compared with the controls was reported (20). 

The aim of this study was therefore to (i) explore the neuroprotective potential of bee venom in an experimental model of PD in mice induced by the pesticide rotenone; (ii) establish a dose-response for bee venom using human-relevant doses; (iii) investigate whether bee venom could alter the therapeutic response to standard antiparkinsonian drugs levodopa/carbidopa and rasagiline; (iv) investigate whether bee venom could be useful “add on” to other antiparkinsonian drugs; (v) examine the utility of using the intradermal route for bee venom in this model instead of acupoint stimulation.

The effect of bee venom either alone or with levodopa/carbidopa or rasagiline was evaluated using biochemical, behavioral, and histopathological measures.

## Materials and Methods

***Animals***

Swiss Albino male mice (weighing 22-25g) were used in the study and kept on forage pellets composed of 7-11% fats and 17-19% proteins (El-Nasr Chemical Company, Cairo, Egypt). Animals received care that followed the recommendations of the Guide for the Care and Use of Laboratory Animals guidelines (National Institutes of Health publication Number 85-23; revised 1996). The study was approved by the Faculty of Pharmacy, Cairo University Research Ethics Committee (approval serial number is PT1214). 

***Drugs and chemicals***

Bee venom (Apis mellifera venom) was purchased from the new technique laboratory LTD (Georgia, USA), HPLC analyzed and its chemical composition was 8% apamin, 18% phospholipase A2, and 62% mellitin. Bee venom was dissolved in isotonic saline solution (0.9% NaCl) and given at doses of (0.065, 0.13, and 0.26 mg/kg, ID). The doses of bee venom in the study were based upon the human dose of Apitox^®^ after conversion to that of mice, according to Page and Barnes conversion tables (21)**.** Rotenone was purchased from Sigma-Aldrich (St Louis, MO, USA) and dissolved in 100% dimethyl sulfoxide (DMSO). Levodopa/carbidopa (25 mg/kg, IP) was purchased from MSD, Pharmaceutical Company (Egypt) and rasagiline (1 mg/kg, IP) was purchased from Inspire Pharma Pharmaceutical Company (Egypt). Any other chemicals and reagents used were of analytical grade and purchased from Sigma-Aldrich (St Louis, MO, USA).

***Experimental design***

Rotenone (1.5 mg/kg, SC) was administered every other day for two weeks and at the same time mice were treated with either the vehicle (DMSO, SC), bee venom (0.065, 0.13, and 0.26 mg/kg, ID), L-dopa/carbidopa (25 mg/kg, IP), L-dopa/carbidopa+bee venom (0.13 mg/kg, ID), rasagiline (1 mg/kg, IP) or rasagiline+bee venom (0.13 mg/kg, ID).

Mice were randomly assigned to nine equal groups (n=12 each):

• Group 1 (Vehicle control): mice were treated with the vehicle (DMSO, SC) once a day every other day for two weeks.

• Group 2 (Rotenone control): mice received rotenone (1.5 mg/kg, ID) once a day every other day for two weeks (22).

• Group 3 (Bee venom): mice were treated with bee venom (0.065 mg/kg, ID), concurrently with rotenone.

• Group 4 (Bee venom): mice were treated with bee venom (0.13 mg/kg, ID), concurrently with rotenone.

• Group 5 (Bee venom): mice were treated with bee venom (0.26 mg/kg, ID) concurrently with rotenone.

• Group 6 (L-dopa/carbidopa): mice received L-dopa/carbidopa (25 mg/kg, ID) concurrently with rotenone.

• Group 7 (L-dopa/carbidopa+Bee venom) mice were given both L-dopa/carbidopa (25 mg/kg, IP) and bee venom (0.13 mg/kg, ID) concurrently with rotenone.

• Group 8 (Rasagiline): mice received rasagiline (1 mg/kg, SC) concurrently with rotenone. 

• Group 9 (Rasagiline+Bee venom): mice were given both rasagiline (1 mg/kg, SC) and bee venom (0.13 mg/kg, ID) concurrently with rotenone.

Twenty-four hours after the last given dose of tested drugs, behavioral testing (wire hanging and staircase tests) were performed. Then, mice were euthanized by decapitation under brief diethyl ether anesthesia, brains were then quickly removed out on an ice-cold plate, both striata were dissected, weighed, and washed with ice-cold phosphate-buffered saline (PBS, pH 7.4) and stored at −80 ^°^C until the different analyses were carried out. The brains of 2 mice per group were placed in 10% neutral-buffered formalin saline for histopathological evaluation and immunohistochemical assessment of caspase-3 expression.

***Biochemical investigations***

*Determination of malondialdehyde content*

Lipid peroxidation was assayed by measuring malondialdehyde (MDA) in the tissue homogenates. The method used was that of Mihara and Uchiyama and Ruiz-Larrea *et al*. (23, 24). Thiobarbituric acid reactive substances react with thiobarbituric acid to produce a pink colored complex with a peak absorbance at 532 nm.

*Determination of nitric oxide content*

Nitric oxide measured as nitrite, the stable end product of nitric oxide radical, was determined by using Griess reagent, according to a known method (25).

*Determination of reduced glutathione content*

Reduced glutathione (GSH) content was measured according to the method of Ellman (26) and modified by Beutler *et al*. and Bulaj *et al*. (27, 28). Ellman’s reagent (5, 5-dithiobis (2-nitrobenzoic acid) or DTNB is reduced to the yellow-colored 2-nitro-5-mercaptobenzoic acid, which has an absorbance of 412 nm.

*Determination of total antioxidant capacity*

A commercial TAC kit from Biodiagnostic (Egypt) was used. The principle is that the antioxidants present in the biological sample react with a known amount of exogenously provided hydrogen peroxide (H_2_O_2_). The residual H_2_O_2_ is determined by an enzymatic reaction that involves the conversion of 3, 5, dichloro -2- hydroxyl benzenesulfonate to a colored product (29).

*Determination of monocyte chemoattractant protein-1*

MCP-1 was estimated by using the MCP-1 enzyme-linked immunosorbent assay (ELISA) kit (Elabscience Biotechnology Co., Ltd) in which the sandwich-ELISA method was used. Standards or samples are added to the appropriate micro ELISA plate pre-coated with an antibody specific to MCP-1 wells and combined with the specific antibody. Then a biotinylated detection antibody specific for MCP-1 and Avidin-Horseradish Peroxidase (HRP) conjugate is added to each microplate well successively and incubated. Free components are washed away. The substrate solution is added to each well. Only those wells that contain MCP-1, biotinylated detection antibody, and Avidin-HRP conjugate will appear blue in color. The enzyme-substrate reaction is terminated by the addition of a sulfuric acid solution and the color turns yellow. The optical density (OD) is measured using a microplate ELISA reader at a wavelength of 450 nm. The OD value is proportional to the concentration of MCP-1.

*Determination of paraoxonase-1 activity*

Arylesterase action of paraoxonase-1 that catalyzes the cleavage of phenylacetate (substrate) into phenols was measured spectrophotometrically at the wavelength of 270 nm in supernatants according to the literature (30, 31).

*Determination of butyrylcholinesterase activity*

The activity of the BChE enzyme was determined using a commercial butyrylcholinesterase kit from BEN, Biochemical Enterprise (Italy). Cholinesterase catalyzes the hydrolysis of butyrilthiocholine (BTC) resulting in the formation of butyrate and thiocholine. Thiocholine reduces hexacyanoferrate (III) into hexacyanoferrate (II). The decrease of absorbance in the unit time at 405 nm is proportional to the activity of the cholinesterase enzyme in the sample.

*Determination of tumor necrosis factor-alpha*

TNF-alpha ELISA kit (RayBiotech, USA) was used for the quantitative measurement of mouse TNF-alpha. This assay employs an antibody specific for mouse TNF-alpha coated on a 96-well plate. Standards and samples are pipetted into the wells and TNF-alpha present in a sample is bound to the wells by the immobilized antibody. The wells are washed and biotinylated anti-mouse TNF-alpha antibody is added. After washing away unbound biotinylated antibody, HRP conjugated streptavidin is pipetted to the wells. The wells are again washed, a TMB substrate solution is added to the wells and color develops in proportion to the amount of TNF-alpha bound. The stop solution changes the color from blue to yellow and the intensity of the color is measured at 450 nm.

*Determination of striatal dopamine content*

According to the mouse dopamine (DA) ELISA kit (CUSABIO, USA), the quantitative sandwich enzyme immunoassay technique used as antibody specific for DA was pre-coated onto a microplate. Standards and samples are pipetted into the wells and any DA present is bound by the immobilized antibody. After removing any unbound substances, a biotin-conjugated antibody specific for DA is added to the wells. After washing, avidin conjugated Horseradish Peroxidase (HRP) is added to the wells. Following a wash to remove any unbound avidin-enzyme reagent, a substrate solution is added to the wells and color develops in proportion to the amount of DA bound in the initial step. The color development is stopped and the intensity of the color is measured.

***Behavioral testing***

*Wire hanging test*

Neuromuscular strength was evaluated using the wire hanging test, where each mouse was placed with its forelimbs on a horizontal wire of 20 cm length, 50 cm above the workbench surface. The latency time that mice took to fall from the wire was recorded; Soft padding was placed on the landing area to avoid injury of the falling mice (32).

*Staircase test*

This test aims to evaluate reaching capability. Mice were placed at the bottom of a stair (30 cm in length) with an angle of 55 ^°^ above the workbench and the latency to climb the stair was recorded for each mouse (33).

*Histological assessment studies*

Brain samples of all animals were dissected immediately after death. The specimens were then fixed in 10% neutral-buffered formalin saline for 72 hr at least. All specimens were washed in tap water for half an hour and then dehydrated in ascending grades of alcohol, cleared in xylene, and embedded in paraffin. 

*Hematoxylin and Eosin staining*

Serial sections of 5 μm thickness were cut and stained with hematoxylin and eosin for histopathological investigation. Images were examined and photographed under a digital camera (Microscope Digital Camera DP70, Tokyo) and processed using Adobe Photoshop version 8.0.

*Immunohistochemical determination of caspase-3*

Paraffin-inserted brain sectors were deparaffinized and hydrated. Immunohistochemistry was done using a mouse monoclonal caspase-3 for detection of the caspase cleavage activity. The paraffin sectors were heated in a microwave oven (25 min at 720 W) for antigen recovery and incubated with either of the anti-caspase antibodies (1:50 dilution) overnight at 4 ^°^C. Then, washed with PBS and incubated with biotinylated goat-anti-rabbit- immunoglobulin G secondary antibodies (1:200 dilution; Dako Corp.) and streptavidin/alkaline phosphatase complex (1:200 dilution; Dako) for 30 min at room temperature, the binding sites of antibody were found with DAB (Sigma). Following washing with PBS, the samples were stained with H&E for 2-3 min and desiccated by transferring them through increasing ethanol solutions (30%, 50%, 70%, 80%, 95%, and 100% ethanol). Following desiccation, the sectors were soaked twice in xylene at room temperature for 5 min, fixed, observed, and assessed by a high-power light microscope.

***Statistical analysis***

Values are expressed as mean±SE. Statistical analysis of the data was done using one-way ANOVA followed by the Duncan test for multiple group comparisons. SPSS (Statistical Package for the Social Sciences) software package was used for data analysis. A probability value of *P<*0.05 was considered statistically significant. Graphs were made using the Graph Pad Prism 6 software program.

## Results

***Effect of bee venom alone or in combination with L-dopa/carbidopa*** ***or rasagiline on rotenone-induced alterations in oxidative stress biomarkers***

Results are shown in Table 1. One way ANOVA indicated a significant treatment effect on MDA (F=16.3, *P<*0.0001), NO (F=11.7, *P<*0.0001), TAC (F=3.63, *P<*0.005), and PON-1 activity (F=9.36, *P<*0.0001). Administration of rotenone significantly increased striatal MDA (*P<*0.05), and NO contents (*P<*0.05) by 31.2% and 168.1%, respectively together with a reduction in GSH and TAC contents and PON-1 activity by 36.6%, 36%, and 52.3% (*P<*0.05 for all) , respectively as compared with the corresponding vehicle control values. 

Bee venom given at 0.065, 0.13, and 0.26 mg/kg resulted in significant decrease in MDA by 20.2%, 29.5%, and 32.9% (*P<*0.05 for all), respectively, while bee venom given at 0.26 mg/kg significantly decreased NO by 27.6% (*P<*0.05) compared with the rotenone control group. Meanwhile, significant and dose-dependent increase in PON-1 activity by 63.6%, 79.3%, and 192.6% (*P<*0.05 for all) was recorded for bee venom treatment at the above doses, respectively, compared with the rotenone control.

Reduced glutathione increased following bee venom administration and reached statistical significance at the dose of 0.26 mg/kg increasing by 42% (*P<*0.05) compared with the rotenone only group. Meanwhile, TAC increased significantly following bee venom at 0.13 or 0.26 mg/kg to 53.1% (*P<*0.05) and 68.7% (*P<*0.05), respectively. Moreover, marked and significant increments in PON-1 activity were observed following treatment with bee venom at 0.065, 0.13, and 0.26 mg/kg by 63.6%, 79.3%, and 92.6% (*P<*0.05 for all), respectively, compared with the rotenone value (Table 1).

In rotenone-treated mice, administration of L-dopa/carbidopa resulted in significantly decreased MDA by 41.7% (*P<*0.05) compared with the rotenone control value. The bee venom/L-dopa/carbidopa combination resulted in significantly lower NO level compared with that of L-dopa alone and significantly increased GSH content to 43.4% (*P<*0.05) compared with the rotenone control group. Also, treatment with L-dopa/carbidopa alone or in combination with bee venom resulted in a significant increase in PON-1 activity by 70% (*P<*0.05) and 121.9% (*P<*0.05) respectively, compared with the rotenone control value.

The administration of rasagiline or bee venom/rasagiline combination to rotenone-treated mice resulted in a significant decrease in MDA content by 20.1% (*P<*0.05) and 38.3% (*P<*0.05), respectively, compared with the rotenone control value. The combination of bee venom with rasagiline resulted in significantly increased GSH and TAC by 33.9% (*P<*0.05) and 81.3% (*P<*0.05), respectively, compared with the rotenone control. There was also significant increase in PON-1 activity by rasagiline treatment or bee venom/rasagiline combination by 60.9 % (*P<*0.05) and 133.3% (*P<*0.05), respectively, compared with rotenone only group (Table 1).

***Effect of bee venom alone or in combination with L-dopa/carbidopa*** ***or rasagiline on rotenone-induced alterations in striatal MCP-1***

One-way ANOVA revealed a significant effect by treatment (F=17.39, *P<*0.0001). Rotenone treatment elevated striatal MCP-1 concentration by 52.7% (*P<*0.0001) as compared with the vehicle control value. Bee venom given at doses of 0.065, 0.13, and 0.26 mg/kg resulted in significantly decreased striatal MCP-1 content by 34.5%, 48.8% and 54.8% (*P<*0.05 for all), respectively. L-dopa/carbidopa or rasagiline given alone or in combination with bee venom at 0.13 mg/kg significantly reduced MCP-1 content by 52.4-44% (*P<*0.05) and 23.8-29.8% (*P<*0.05), respectively, compared with the rotenone control value (Table 2).

***Effect of bee venom alone or in combination with L-dopa/carbidopa (25 mg/kg, IP)*** ***or rasagiline (1 mg/kg, IP) on rotenone-induced alterations in striatal cholinesterase***

There was a significant effect by treatment (F=18.63, *P<*0.001). Rotenone treatment reduced BuChE activity by 27.3% (*P<*0.05) as compared with the vehicle control value. Striatal BuChE showed significant increase by 61.8%, 40.0% and 179.7% (*P<*0.05 for all) following treatment with bee venom at doses 0.065, 0.13 and 0.26 mg/kg, respectively as compared with the rotenone control. Normalization of BuChE activity was observed after treatment with bee venom at 0.065 and 0.13 mg/kg. 

L-dopa/carbidopa or rasagiline resulted in significant increase in BuChE activity by 57.8% (*P<*0.05) and 219.8% (*P<*0.05), respectively. Meanwhile, the combined treatment of bee venom/L-dopa/carbidopa or bee venom/rasagiline caused significantly increased BuChE activity by 131.8% (*P<*0.05), and 133.8% (*P<*0.05), respectively, compared with the rotenone control value. The bee venom/L-dopa/carbidopa combination was statistically significant from bee venom at 0.13 mg/kg or L-dopa/carbidopa alone in increasing BuChE activity. The rasagiline/bee venom combination was significantly better than bee venom alone while rasagiline alone was significantly better than rasagiline/bee venom combination (Table 3).

***Effect of bee venom either alone or in combination with rasagiline on rotenone-induced alterations in striatal tumor necrosis factor-alpha (TNF-alpha)***

**Figure 1:**
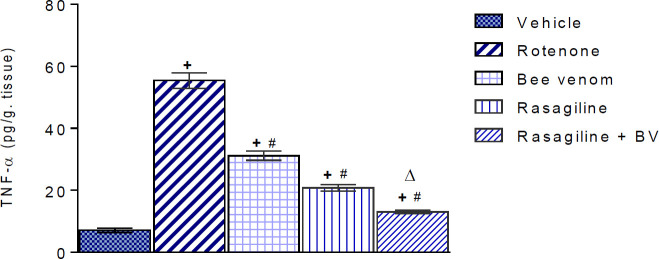
Effects of bee venom (0.13 mg/kg, ID), rasagiline (1 mg/kg, IP), and their combination on rotenone-induced alterations in striatal tumor necrosis factor-alpha (TNF-alpha) content

One-way ANOVA showed a significant treatment effect (F=99.36, *P<*0.001). Compared with the vehicle-treated group, rotenone markedly increased the level of the pro-inflammatory cytokine TNF-alpha in the striatum by 680.3% (55.4 ± 2.5 vs 7.1 ± 0.67 pg/g. tissue, *P<*0.05). Bee venom, rasagiline, and their combination reversed this effect and striatal TNF-alpha decreased by 43.7%, 62.5, and 76.4% (*P<*0.05 for all), respectively, compared with the rotenone control. Values are 31.18±1.49, 20.8±1.0, and 13.05±0.61 pg/g. tissue for bee venom, rasagiline, and bee venom/rasagiline groups, respectively (Figure 1).

***Effect of bee venom either alone or in combination with rasagiline on rotenone-induced alterations in striatal dopamine (DA) content***

**Figure 2:**
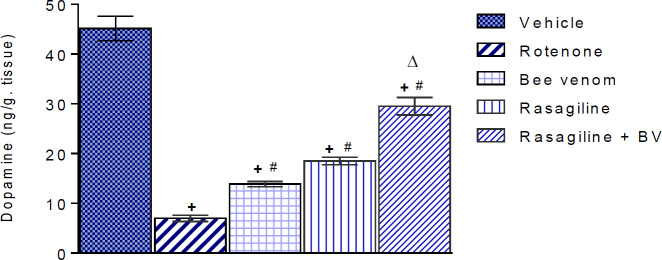
Effects of bee venom (0.13 mg/kg, ID), rasagiline (1 mg/kg, IP), and their combination on rotenone-induced alterations in striatal dopamine (DA) content

There was a significant effect by treatment (F=148.1, *P<*0.0001). Rotenone caused depletion of striatal dopamine by 85.5% (*P<*0.05) compared with the vehicle control group (45.13±2.47 vs 6.98±0.61 ng/g. tissue). Bee venom, rasagiline, and their combined treatment reversed this effect where dopamine content increased by 98.4%, 165.0, and 322.6% (*P<*0.05 for all) of that in the rotenone control group, respectively. Values are 13.85±0.54, 18.5±0.75 and 29.5±1.79 ng/g. tissue for bee venom, rasagiline, and bee venom/rasagiline groups, respectively (Figure 2).

***Effect of bee venom either alone or in combination with L-dopa/carbidopa or rasagiline on rotenone-induced behavioral alterations***

*Wire hanging test*

There was a significant effect by treatment (F=3.67, *P=*0.016). Rotenone treatment led to a significant decrease in the time the mice spent suspended from a wire by 46.5% (*P<*0.05) compared with the vehicle control value (19.17±1.21 vs 35.84±1.54 sec). The administration of bee venom at doses 0.065, 0.13, and 0.26 mg/kg resulted in significant increase in the time spent by 182.5%, 404.3%, and 280.1% (*P<*0.05 for all), respectively, compared with the rotenone group. Treatment with L-dopa/carbidopa, or rasagiline given alone or in combination with bee venom (0.13 mg/kg) resulted in a marked significant increase in the time spent to 130.4-343.4%, and 182.6-260% (*P<*0.05 for all), respectively, compared with the rotenone control value.

**Figure 3:**
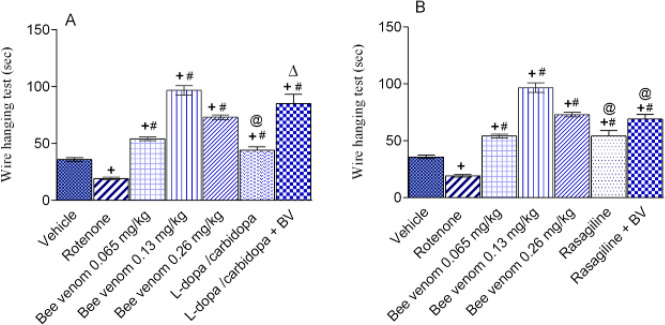
Effect of bee venom either alone or in combination with L-3, 4-dihydroxyphenylalanine (L-dopa)/carbidopa or rasagiline (1 mg/kg, IP) on rotenone-induced behavioral wire hanging test alterations

Bee venom alone was statistically significant in increasing the ability of mice to hang to the wire compared with L-dopa/carbidopa or rasagiline alone and from rasagiline in combination with bee venom. Meanwhile, L-dopa /bee venom combination was statistically significant from L-dopa/carbidopa in increasing the ability of mice to hang to the wire (Figures 3A and 3B).

*Staircase test*

There was a significant treatment effect (F=6.31, *P<*0.0001). The time spent to ascend the inclined stair was significantly increased by 67.7% (*P<*0.05) in rotenone-treated mice compared with the vehicle-treated group (33.73±2.0 vs 20.11±1.37 sec). Bee venom, rasagiline, and their combination normalized the time recorded in the stair test.

**Figure 4:**
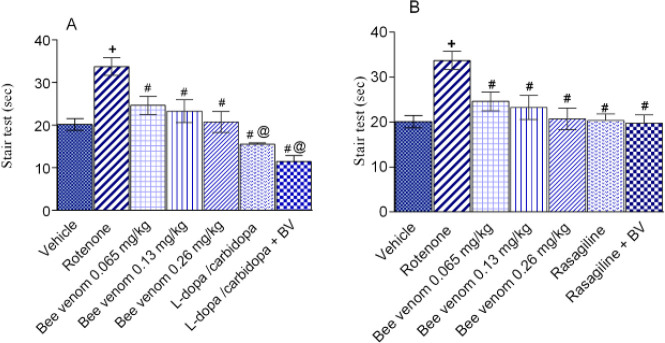
Effect of bee venom either alone or in combination with L-3, 4-dihydroxyphenylalanine (L-dopa)/carbidopa (25 mg/kg, IP) or rasagiline (1 mg/kg, IP) on rotenone-induced behavioral staircase test alterations

Bee venom at doses 0.065, and 0.13 mg/kg decreased the time recorded in stair test by 27.1% (*P<*0.05), and 31.1% (*P<*0.05), respectively, compared with that of the rotenone control. Bee venom given at 0.26 mg/kg normalized the time recorded in the stair test. The administration of L-dopa/carbidopa or rasagiline alone or in combination with bee venom (0.13 mg/kg) resulted in a significant decrease in the recorded time by 54-66.1% and 40.0-41.3% (*P<*0.05 for all), respectively, compared with the rotenone control (Figures 4A and 4B).

***Effect of bee venom either alone or in combination with L-dopa/carbidopa*** ***or rasagiline on rotenone-induced histopathological alterations ***

**Figure 5:**
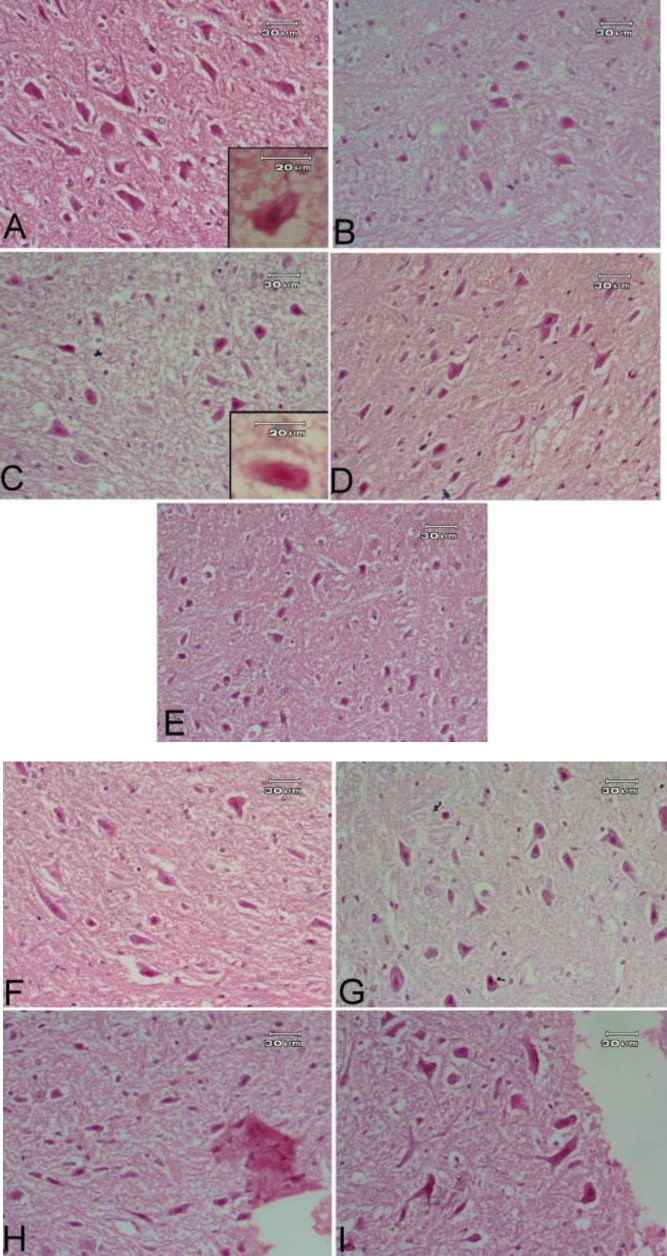
Effect of bee venom either alone or in combination with L-3, 4-dihydroxyphenylalanine (L-dopa)/carbidopa or rasagiline on rotenone-induced striatal histopathological alterations using hematoxylin/eosin stain

Administration of rotenone results in a marked decrease in the number and size of striatal neurons. Treatment with bee venom ameliorated the reduction in neuronal size and number in a dose-dependent manner. Moreover, treatment with rasagiline alone and in combination with bee venom showed a moderate increase in neuronal size and number (Figure 5).

***Effect of bee venom either alone or in combination with L-dopa/carbidopa or rasagiline on rotenone-induced caspase-3 expression***

**Figure 6:**
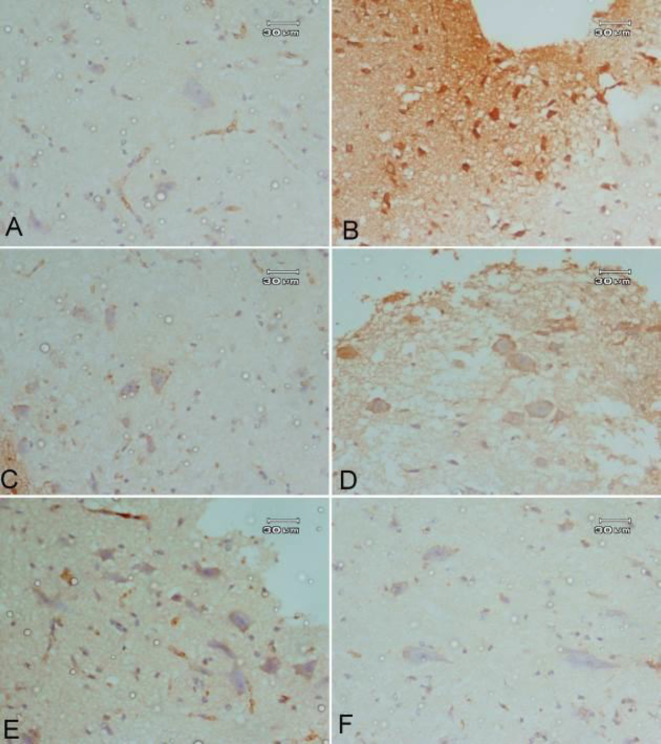
Effect of bee venom either alone or in combination with L-3, 4-dihydroxyphenylalanine (L-dopa)/carbidopa or rasagiline on rotenone-induced caspase-3 expression. A photomicrograph of a substantia nigra area stained immunohistochemically with the caspase-3 antibody of (A) Vehicle-control mice shows a negative result in the substantia nigra area. (B) Rotenone (1.5 mg/kg, SC) control-treated mice show multiple neurons that are strongly positive stained. Notice the neurons are small in size. (C) Rotenone and bee venom (0.13 mg/kg, ID) treated mice show only a few neurons with a positive result for the stain, while most of the neurons are of weak stain result. (D) Rotenone, L- dopa/carbidopa (25 mg/kg, IP) and bee venom (0.13 mg/kg, ID) stained immunohistochemically with caspase-3 antibody shows weak positive stain in most of the neurons. Almost all neurons regain their normal size. (E) Rotenone and rasagiline (1 mg/kg, IP) treated mice show positive result in many of the neurons. (F) Rotenone, rasagiline (1 mg/kg, IP), and bee venom (0.13 mg/kg, ID) show a very weak positive result in a few neurons, while most of the neurons show negative results

Brain sections stained with caspase-3 antibody in rotenone-treated mice showed high caspase-3 expression where multiple neurons were positively stained and the neurons were small in size when compared with a photomicrograph of a brain section of vehicle control mice which showed a negative result in the substantia nigra area. Treatment with bee venom showed only a few neurons with a positive result for the stain in the substantia nigra area. Rasagiline-treated mice showed a positive result in many of the neurons, while combined treatment showed a very weak positive result in only a few neurons (Figure 6).

**Table 1:** Effect of bee venom alone or in combination with L-dopa/carbidopa or rasagiline on rotenone-induced alterations in oxidative stress biomarkers

**Group**		**MDA** **(nmol/g. tissue)**	**NO** **(µmol/g. tissue)**	**GSH** **(** **µmol** **/g.tissue)**	**TAC** **(µmol/g.tissue)**	**PON1** **(kU/L)**
**Vehicle**		35.86±1.48	25.26±3.84	4.65±0.13	0.50±0.04	13.09±1.17
**Rotenone (1.5 mg/kg)**		47.06±1.16^+^	67.71±6.02^+^	2.95±0.08^+^	0.32±0.02^+^	6.24±0.39^+^
**Rotenone 1.5mg/ kg**	**Bee venom** **0.065 mg/kg**	37.55±1.13^#^	62.84±4.69	2.88±0.35	0.32±0.002	10.21±0.34^#^
	**Bee venom** **0.13 mg/kg**	33.19±0.86^#^	58.53±2.92	3.32±0.09	0.49±0.01^#^	11.19±1.35^#^
	**Bee venom** **0.26 mg/kg**	31.56±0.82^#^	49.05±3.47^#^	4.19±0.09^#^	0.54±0.02^#^	18.26±1.74^#^
	**L-dopa /carbidopa ** **(25 mg/kg, IP)**	27.45±1.4^#$α^	73.17±4.51^$α^	2.77±0.1^$α^	0.27±0.03^$^	10.61±1.14^#^
	**L-dopa/carbidopa (25 mg/kg, IP) ** **+ Bee venom** ** (0.13 mg/kg, ID)**	33.59±1.81^#^	58.36±4.11	4.23±0.17^#$^	0.30±0.03^$^	13.84±1.43^#^
	**Rasagiline** **(1 mg/kg, IP)**	37.61±1.39^#Δ^	57.74±3.24	3.20±0.15^Δ^	0.24± 0.03^$Δ^	10.04±0.87^#Δ^
	**Rasagiline** **(1mg/kg, IP) ** **+ Bee venom (0.13 mg/kg, ID)**	29.02±0.99^#^	54.59±2.93	3.95±0.13^#$^	0.58±0.02^#^	14.56±1.36^#^

All treatments were administered every other day for 15 days concurrently with rotenone (1.5 mg/ kg, SC)

Values are expressed as means±SE (n=12). The data were analyzed by one-way analysis of variance (ANOVA) followed by Duncan’s multiple range test+indicates significant difference from the DMSO group; # indicates significant difference from the rotenone group; $ indicates significant difference from the bee venom (0.13 mg/kg) group; α indicates significant difference from L-dopa/carbidopa+bee venom (0.13 mg/kg) group; Δ indicates significant difference from rasagiline+bee venom (0.13 mg/kg) group

**Table 2:** Effect of bee venom alone or in combination with L-dopa/carbidopa or rasagiline on rotenone-induced alterations in striatal MCP-1

**Group**		**MCP-1 (pg/mL)**
**Vehicle**		0.55±0.04
**Rotenone (1.5 mg/kg)**		0.84±0.04^+^
**Rotenone ** **1.5 mg/ kg**	**Bee venom 0.065 mg/kg**	0.55±0.05^#^
	**Bee venom 0.13 mg/kg**	0.43±0.02^#^
	**Bee venom 0.26 mg/kg**	0.38±0.04^#^
	**L-dopa /carbidopa (25 mg/kg, IP)**	0.40±0.04^#^
	**L-dopa/carbidopa (25 mg/kg, IP) ** **+ Bee venom (0.13 mg/kg, ID)**	0.47±0.04^#^
	**Rasagiline (1 mg/kg, IP)**	0.64±0.06^#$^
	**Rasagiline (1 mg/kg, IP) ** **+ Bee venom (0.13 mg/kg, ID)**	0.59±0.02^#$^

All treatments were administered every other day for 15 days concurrently with rotenone. Data are expressed as means±SE (n=12)The data were analyzed using one-way analysis of variance (ANOVA) followed by Duncan’s multiple range test

+ indicates significant difference from the vehicle control group; # indicates significant difference from the rotenone control group; $ indicates significant difference from the bee venom (0.13 mg/kg) group

**Table 3:** Effect of bee venom alone or in combination with L-dopa/carbidopa or rasagiline on rotenone-induced alterations in striatal cholinesterase

**Group**		**BuChE** **(U/L)**
**Vehicle**		223.46 ± 11.48
**Rotenone (1.5 mg/kg)**		162.37 ± 8.7^+^
**Rotenone ** **1.5 mg/ kg**	**Bee venom 0.065 mg/kg**	262.78± 14.43^#^
	**Bee venom 0.13 mg/kg**	227.1 ± 15.77^#^
	**Bee venom 0.26 mg/kg**	454.20± 7.86^#^
	**L-dopa /carbidopa (25 mg/kg, IP)**	256.29± 9.95^#α^
	**L-dopa/carbidopa (25 mg/kg, IP) ** **+ Bee venom (0.13 mg/kg, ID)**	376.33± 16.20^#$^
	**Rasagiline (1 mg/kg, IP)**	519.23± 13.405^Δ^
	**Rasagiline (1 mg/kg, IP) ** **+ Bee venom (0.13 mg/kg, ID)**	379.58± 18.41^#$^

All treatments were administered every other day for 15 days concurrently with rotenone. Data are expressed as means±SE (n=12)The data were analyzed using one-way analysis of variance (ANOVA) followed by Duncan’s multiple range test+indicates significant difference from the vehicle control group; # indicates significant difference from the rotenone control group; $ indicates significant difference from bee venom; α indicates significant difference from L-dopa/carbidopa+bee venom (0.13 mg/kg) group; Δ indicates significant difference from rasagiline+bee venom (0.13 mg/kg) group

All treatments were administered every other day for 15 days concurrently with rotenone (1.5 mg/ Kg, SC). Values are expressed as means±SE (n=12). The data were analyzed by one-way analysis of variance (ANOVA) followed by Duncan’s multiple range test + indicates significant difference from the vehicle control group; # indicates significant difference from the rotenone control group; Δ indicates significant difference from rasagiline group

All treatments were administered every other day for 15 days concurrently with rotenone (1.5 mg/ Kg, SC). Values are expressed as means±SE (n=12). The data were analyzed using one-way analysis of variance (ANOVA) followed by Duncan’s multiple range test; + indicates significant difference from the vehicle control group; # indicates significant difference from the rotenone control group; Δ indicates significant difference from rasagiline group

All treatments were administered every other day for 15 days concurrently with rotenone (1.5 mg/ kg, SC). Values are expressed as means±SE (n=12). The data were analyzed by one-way analysis of variance (ANOVA) followed by Duncan’s multiple range test (*P<*0.05) and Graph Pad Prism 6 software program+indicates significant difference from the vehicle group # indicates significant difference from the rotenone control group @ indicates significant difference from bee venom (0.13 mg/kg) group Δ indicates significant difference from L-dopa/carbidopa group

All treatments were administered every other day for 15 days concurrently with rotenone (1.5 mg/ Kg, SC). Values are expressed as means±SE (n=12). The data were analyzed by one-way analysis of variance (ANOVA) followed by Duncan’s multiple range test ; + indicates significant difference from the vehicle group; # indicates significant difference from the rotenone control group; @ indicates significant difference from bee venom (0.13 mg/kg) group

A photomicrograph of sections of substantia nigra area in the brain of (A) Vehicle-control mice shows the normal appearance of pigmented neurons in this area. (B) Rotenone (1.5 mg/kg SC) control-treated mice shows a marked decrease in the number and size of neurons. (C) Rotenone and bee venom (0.065 mg/kg, ID) treated mice show mild amelioration in neurons’ size, although their number is still less than normal. (D) Rotenone and bee venom (0.13 mg/kg, ID) treated mice shows remarkable amelioration in neurons’ size and number. (E) Rotenone and bee venom (0.26 mg/kg, ID) treated mice show a moderate increase in neurons’ number, but most of them are smaller in size than normal. (F) Rotenone and L- dopa/carbidopa (25 mg/kg, IP) treated mice show only some neurons regain their normal size, while most of them are smaller than normal. (G) Rotenone, L- dopa/carbidopa and bee venom (0.13 mg/kg, ID) treated mice show most of the neurons appear of nearly normal size and some of them are still smaller than normal. (H) Rotenone and rasagiline (1 mg/kg, IP) treated mice show most neurons are smaller in size than normal. (I) Rotenone, rasagiline and bee venom (0.13 mg/kg, ID) treated mice show a marked increase in the size of most of the neurons

## Discussion

The findings of this study indicate that the systemic administration of rotenone, a pesticide, and a mitochondrial complex I inhibitor to mice resulted in increased striatal oxidative stress indicated by the increase in the lipid peroxidation marker and depletion of the antioxidants GSH, TAC, and PON-1. Rotenone also increased striatal MCP-1, TNF-α, apoptosis (caspase-3 activation), caused nigrostriatal neurodegeneration, and hypokinetic motor deficits. These biochemical, behavioral, and histopathological changes induced by rotenone were ameliorated following treatment with bee venom at clinically relevant doses. When given in combination with the standard antiparkinsonian drugs L-dopa/carbidopa or rasagiline, bee venom rather increased their neuroprotective effects. Our study, therefore, provides further support to bee venom as a potential neuroprotective agent in PD either alone or as “add on” therapy.

In the present study, rotenone administration induced Parkinson′s disease-like behavioral, biochemical, and histopathological changes; an effect that was previously reported (34-37) (19). Rotenone induces apoptosis through inhibiting the mitochondrial respiratory chain and so raises reactive oxygen species which promotes cell death and is a critical pathological factor in neurodegenerative diseases accompanied by apoptotic features as mitochondrial damage, microglial activation, oxidative damage, dopaminergic degeneration, and L-dopa-responsive motor deficit. Moreover, rotenone-induced activation of the caspase-3 pathway and increased levels of caspase family gene expression contribute to apoptosis (37, 19). In the present study, the administration of rotenone increased brain MDA along with significant decrements in the antioxidant GSH and TAC. Our results are therefore in agreement with the work of different studies that demonstrated increased lipid peroxidation and decreased antioxidants such as the level of reduced glutathione (GSH) and the activity of paraoxonase 1 (PON1) in the rodent brain following rotenone injection (38-39). Moreover, researchers have previously shown that rotenone is capable of causing caspase3/9-induced apoptosis in a rat model of MPTP or rotenone-induced PD (40). Our findings also indicate decreased striatal PON-1 in the striata of rotenone-treated mice. The PON-1 enzyme which belongs to a family comprising PON-1, PON-2, and PON-3 and acts to hydrolyze organophosphorus insecticides (41) derives its importance from the evidence linking variation in its activity with the increased risk for developing PD (42). Other researchers reported marked inhibition of PON-1 and BuChE activities in the rat brain after systemic rotenone injection, possibly due to increased tissue oxidative stress by the toxicant (43).

Bee venom is composed of polypeptides, enzymes, amines, lipids, and amino acids (44). It has nociceptive and neurotoxic effects, recently, it was demonstrated to have radioprotective (45), antimutagenic (46), anti-inflammatory (47), antinociceptive (48), and anticancer potentials (49). Phospholipase A_2_ (PLA_2_), one of the enzymes present in bee venom, has a protective efficiency against different diseases, including arthritis, asthma, PD, and drug-induced organ inflammation (50, 51).

Our results demonstrate that the rotenone induced-behavioral dysfunction was effectively improved by bee venom which was able to reverse the motor deficits in the wire hanging and staircase tests. These tests measure motor strength and locomotor abilities, respectively (32, 33), and have been shown to be markedly impaired after rotenone injection in rodents (39). Using bee venom (1.0 mg/kg every 24 hr, for 6 days, IP), a study reported improvements in the impaired motor performance in the horizontal bar and open field tests in the rotenone model of PD (52). Using the 6-hydroxydopamine-induced PD in rats, research found that bee venom (at 1 or 3 μg/kg, IP, every 3 days) was able to ameliorate contralateral forelimb akinesia and the rotations induced by the dopaminergic agent apomorphine. Catalepsy caused by haloperidol, a dopamine D2 receptor antagonist was also prevented by a single injection of bee venom (18). The pharmacological efficacy of bee venom may be due to its PLA_2_ which suppresses MPTP-induced neurotoxicity and oxidative stress in the nigrostriatal system (53). Moreover, different studies showed that treatment with bee venom PLA_2_ resulted in significant restoration of motor dysfunction in PD mice through lowering alpha-Syn; a presynaptic neuronal protein that is linked genetically and neuropathologically to PD (54-56).

Additionally, the bee venom-induced neuroprotection in the current work may be secondary to inhibition of oxidative stress as evidenced by the reduction in lipid peroxides, nitric oxide, and elevation of total antioxidant capacity and both glutathione and PON1 activities. The antioxidant efficacy of BV had been confirmed in several pathological studies (57). Moreover, a study reported increased antioxidants as glutathione (GSH) (total and reduced), after bee venom treatment subcutaneous injection with BV in a dose of 0.01, 0.05, and 0.1 mg/kg, respectively in rats (58).

Accumulated evidence indicates that PD is associated with enhanced inflammatory response and damage (59). This is in line with observed enhancement in MCP-1 and TNF-alpha contents in the rotenone-treated group, an effect that was ameliorated with bee venom, in the present investigation. Another study using the MPTP-induced PD in mice found that bee venom rescued nigrostriatal dopaminergic cells (17). The authors attributed this neuroprotective effect to deactivation of microglial cells as well as to a decrease in T cell infiltration, thereby decreasing neuroinflammation. The effect of bee venom on neuroinflammation might be due to the presence of melittin, one of the main active components of bee venom, it is a peptide with diverse therapeutic activities, including antimicrobial, antitumor, and anti-inflammatory effects (60). In this context, Darwish *et al*. found that bee venom acupuncture (0.5 mg/kg, SC) was able to inhibit the inflammatory response by inhibiting TNF-α and NF-κB in rat brain in a model of methotrexate-induced hepatotoxicity (61). Moreover, a group reported that bee venom mediates anti-inflammatory, anti-arthritic, and neuroprotective effects and may represent an anticancer therapeutic to cancer cells after treatment with melittin, a bee venom component (62).

In the current investigation bee venom successfully protected dopaminergic neurons and increased striatal dopamine in rotenone treated-mice which is in line with researchers who illustrated that bee venom stopped the loss of dopaminergic neurons in the striatum and substantia nigra (9). Bee venom prevented the death of dopaminergic NSC34 motor neuron cells induced by rotenone *in vitro* (19). Stimulation of midbrain dopaminergic neurons may be due to the presence of bee venom toxin apamin, the only polypeptide neurotoxin of bee venom that passes the blood-brain-barrier and irreversibly blocks SK channels that is present on nigral dopaminergic neurons and controls their firing pattern and survival, thus enhances cognitive performance, reverses motor deficits, and attenuates anxiety-cognitive-related behavior (63, 64)**. **Moreover, the imbalance between dopaminergic and cholinergic neurotransmission is a hallmark of PD. Therefore, anticholinergic drugs such as atropine are still in use for treatment of PD (65). In the present study, bee venom showed anticholinergic property as it increased striatal BuChE enzyme activity.

The protective effect of bee venom in this model of experimental PD was also confirmed by histological results that revealed the amelioration of both size and number of neurons and inhibition of caspase-3 expression in neuronal cells. Bee venom therapy is considered a psycho-neurological progress in autoimmune and nervous system diseases as bee venom attenuates neuro-inflammatory events by enhancing cell viability and ameliorates mitochondrial impairment and so reduces microglial activation in various neurological disease models (66, 19). A study reported that BV repairs the damaged tissue by decreasing the protease activity and the level of reactive oxygen species (67). Bee venom stops apoptotic pathways, which is verified by reduced DNA fragmentation and inhibited caspase-3 activation (68).

## Conclusion

Our study suggests that bee venom therapy either alone or in combination with standard antiparkinsonian drugs might prove of value in reducing neurodegeneration and in relief of symptoms accompanying this neurodegenerative disease.
